# Radiological assessment of Sella Turcica morphology correlates with skeletal classes in an Austrian population: an observational study

**DOI:** 10.1007/s11282-024-00785-z

**Published:** 2024-11-13

**Authors:** Julia Schwab, Lars Stucki, Sebastian Fitzek, Aliza Tithphit, Andreas Hönigl, Sarah Stackmann, Ina Horn, Hanna Thenner, Philipp Dasser, Ramona Woitek, Kyung-Eun Choi, Sepideh Hatamikia, Julia Furtner

**Affiliations:** 1 Faculty of Medicine and Dentistry, Danube Private University, Krems, Austria; 2 Research Center for Medical Image Analysis and Artificial Intelligence, Department of Medicine, Danube Private University, Krems, Austria; 3 Department of Prosthodontics and Biomaterials, Faculty of Medicine and Dentistry, Danube Private University, Krems, Austria; 4 Research Center for Clinical AI-Research in Omics and Medical Data Science (CAROM), Department of Medicine, Danube Private University, Krems, Austria; 5 Austrian Center for Medical Innovation and Technology, (ACMIT), Wiener Neustadt, Austria; 6 Division of Oral Surgery and Orthodontics, Department of Dental Medicine and Oral Health, Medical University of Graz, Graz, Austria; 7 Faculty of Medicine, Johannes Kepler University, Linz, Austria

**Keywords:** Sella turcica, Dental radiography, Skull base, Orthodontics, Austria

## Abstract

**Objectives:**

This study aimed to analyze variations in the sella turcica (ST) concerning its size, shape, and bridging, providing first reference values in Austrian individuals. Additionally, it assessed associations between these morphological and demographic parameters and their correlation with patients’ skeletal class.

**Methods:**

208 lateral cephalometric radiographs (154 female, 54 male; age 8–58 years) from DPU Dental Clinic (Austria) were included. Size, skeletal class, shape, age, and gender of ST were tested for significance in correlation using, (M)ANOVA, and chi-square.

**Results:**

Linear dimensions of ST ranged from 11.1 to 12.9 mm across readers, with a standard deviation of 2.0–2.2 mm. Normal ST (49.76%) and round ST (58.77%) were the most frequent. ST bridging was detected in 6.97%. Skeletal class I appeared most frequently (54.8%). Statistical significance was observed between age, gender, and ST length, with further significant age effects on ST shape. Moreover, age showed significant modification of ST shape, while skeletal parameters appeared unaffected by other ST parameters.

**Conclusions:**

These preliminary findings define normal ST dimensions in an Austrian population, offering reference values for clinical interpretation and broadening the available European data. Clear associations between morphological and demographic parameters were detected. Additionally, these findings may contribute to diagnostic and therapeutic strategies in orthodontics and craniofacial pathology. Future studies employing cone beam computed tomography (CBCT) along a larger sample size could enhance the generalizability of these findings.

## Introduction

The sella turcica (ST) is a key cephalometric reference in orthodontic treatment planning. It provides a stable point for analyzing craniofacial growth patterns and skeletal relationships in diagnosing and treating malocclusions [1]. Since the ST encloses the pituitary gland, its shape and size are significantly influenced by the development of the gland itself. Therefore, deviations in ST shape and size can indicate pituitary conditions like tumors or hypoplasia [2–6]. Early detection of these deviations is crucial, as pituitary tumors can lead to loss of vision or even be life-threatening [7]. To interpret the ST on dental radiographs, knowledge of normal ST dimensions and shapes is required. However, a recent meta-analysis reveals high variability in size and shape among the general population, lacking Austrian data. The normal sella shape was found to be the most common, occurring in 55.56%, followed by the ST bridge at 11.34%, irregularities in the posterior part of the dorsum sellae at 9.74%, an oblique anterior wall at 9.55%, a double contour of the floor at 6.89%, and a pyramidal shape of the dorsum sellae at 6.6% [8]. Additionally, Iskra et al. determined that the ST has a mean length of 9.06 mm, depth of 8 mm, and diameter of 11.15 mm. Nonetheless, size and shape appear to be influenced by several factors, including ethnicity, age, and gender [2]. For instance, North Americans exhibit the highest and Europeans lowest values in length, and Asians show a larger ST area compared to Europeans [2, 9, 10].

Given the pituitary gland’s significant role in body growth, the size of the ST might reflect certain growth patterns, which are evident in different skeletal angle classes. Specifically, patients with skeletal class II seem to have smaller ST diameters and depths compared to those with other skeletal classes [2, 11, 12].

This study aims to analyze the morphology and dimensions of the ST in lateral cephalometric radiographs from an Austrian orthodontic patient cohort. The goal is to establish normal sex- and age-related reference values for sella parameters and to investigate their relationship to demographic and skeletal factors.

## Materials and methods

This retrospective analysis included all patients who received lateral cephalometric radiographs for diagnostic or therapeutic purposes at the Danube Private University Dental Clinic from December 2015 to February 2023. The study received ethical approval from the Danube Private University Commission on Research Integrity and Ethics (DPU-EK/031).

### Patient selection

A total of 256 lateral cephalometric radiographs were initially retrieved from the patients’ chart system. However, 48 cephalometric radiographs had to be excluded due to image artifacts or poor image quality. No radiographs were excluded due to ST-changing syndromes or tumors in the patients' medical history. The final sample included 154 females and 54 males, resulting in 208 patients being included in the analysis. The age of the selected group ranged between 8 and 58 years.

Demographic data from the Austrian National Statistics Institute via STATcube were evaluated to ascertain the nationality and country of birth, demonstrating that in 2024, 95% of the Austrian population held European passports, with this figure reaching 97% in the province of Lower Austria. Furthermore, it was established that 93% of the Austrian population were born in Europe, excluding Turkey and Russia. Based on these results, it was assumed that the cohort was predominantly European [13].

### Sample size determination

The sample size for this study was determined based on the availability of high-quality radiographs from the target population. Although a formal power analysis was not conducted prior to data collection, we performed a post hoc power analysis to assess the statistical power of the study in detecting significant differences. The post hoc analysis revealed a power of approximately 58.5%, which indicates that the study is sufficiently powered to detect medium effect sizes. While this level of power suggests that some caution should be exercised when interpreting the findings, it is adequate to support the preliminary conclusions drawn about the morphological characteristics of the sella turcica in this cohort. These results provide an important foundation for future research with larger sample sizes, where a formal power analysis will be conducted to further validate and expand upon these findings.

### Measurements of the Sella *Turcica*

**Fig. 1:**
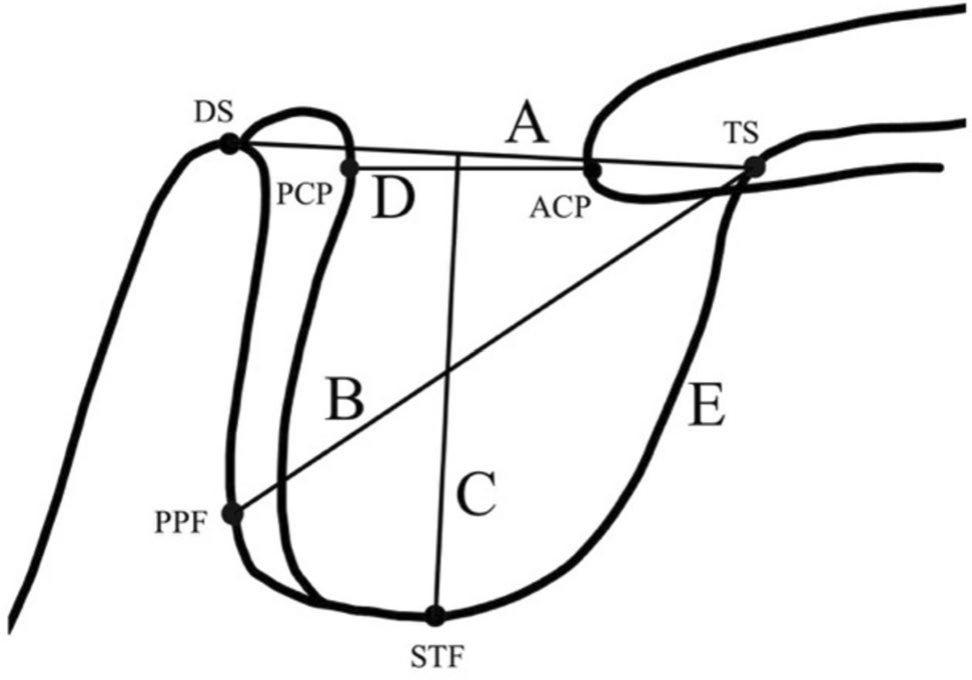
Anatomical reference points used for measuring the dimension of the ST. *DS* dorsum sellae, *TS* tuberculum sellae, *STF* sella turcica floor, *PPF* posterior inner wall of pituitary fossa, *PCP* posterior clinoid process, *ACP* anterior clinoid process, **A** STL (mm) sella turcica length, **B** 4. STDi (mm) sella turcica diameter, **C** STDe (mm) sella turcica depth, **D** ID (mm) interclinoidal distance, **E** STP (mm) sella turcica perimeter

Measurements were conducted using the ImageJ software (version 1.8.0, NIH, Bethesda, MD), which is a Java-based open-source image processing software program. Measurements were taken directly from a monitor. The scale was calibrated precisely according to the millimeter calibration ruler indicated on each lateral cephalogram x-ray. To enhance reliability and precision, the radiological analysis was performed by three independent readers, with one of them conducting a second round of measurements on 100 lateral cephalograms after three months. The background of the readers included an experienced neuroradiologist who trained six final-year dental students (three of whom were measuring ST and three of whom were analysing ST shape). Moreover, unclear or complex cases were supervised by the neuroradiologist. The student group had at least five years of experience and training in interpreting lateral cephalometric radiographs by an oral radiologist and orthodontist. Skeletal classes were radiologically evaluated by a dentist with orthodontic experience. To assess the size of ST, six anatomical reference points were considered according to the methods defined by Silverman and Axelsson [12, 14, 15]. To evaluate the length and depth of the ST, a line was drawn from dorsum sellae (DS) to tuberculum sellae (TS), and a perpendicular line was placed from the former line (DS-TS) to the deepest point of the ST floor (STF), respectively, using the straight tool. Using the same tool, the posterior clinoid process (PCP) and the anterior clinoid process (ACP) were marked to determine the interclinoidal distance, while the diameter of the ST was taken from TS to the furthest point on the posterior inner wall of the pituitary fossa (PPF). The measurement of the perimeter was performed with the polygon selections tool according to the reference points described above. (Fig. 1) shows a graphical representation of the anatomical reference points and the measured dimensions of the ST.

## Shape and Bridging of Sella *Turcica*

**Fig. 2:**
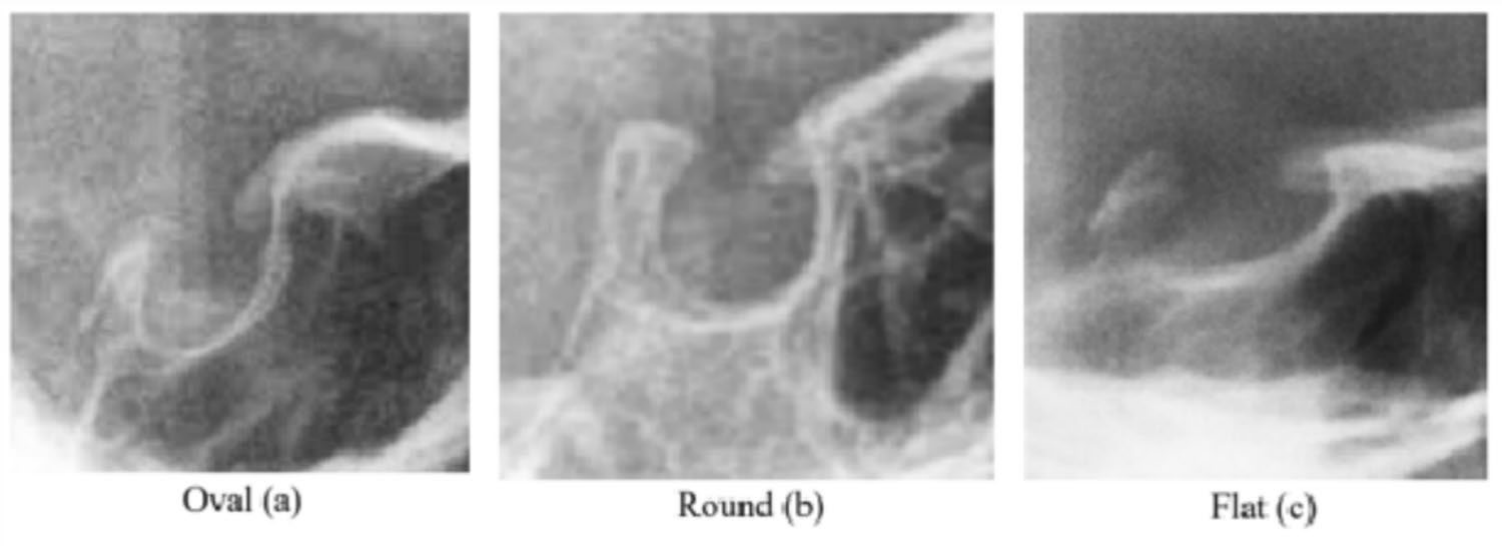
ST shape classified into oval (**a**), round (**b**) and flat (**c**)

**Fig. 3:**
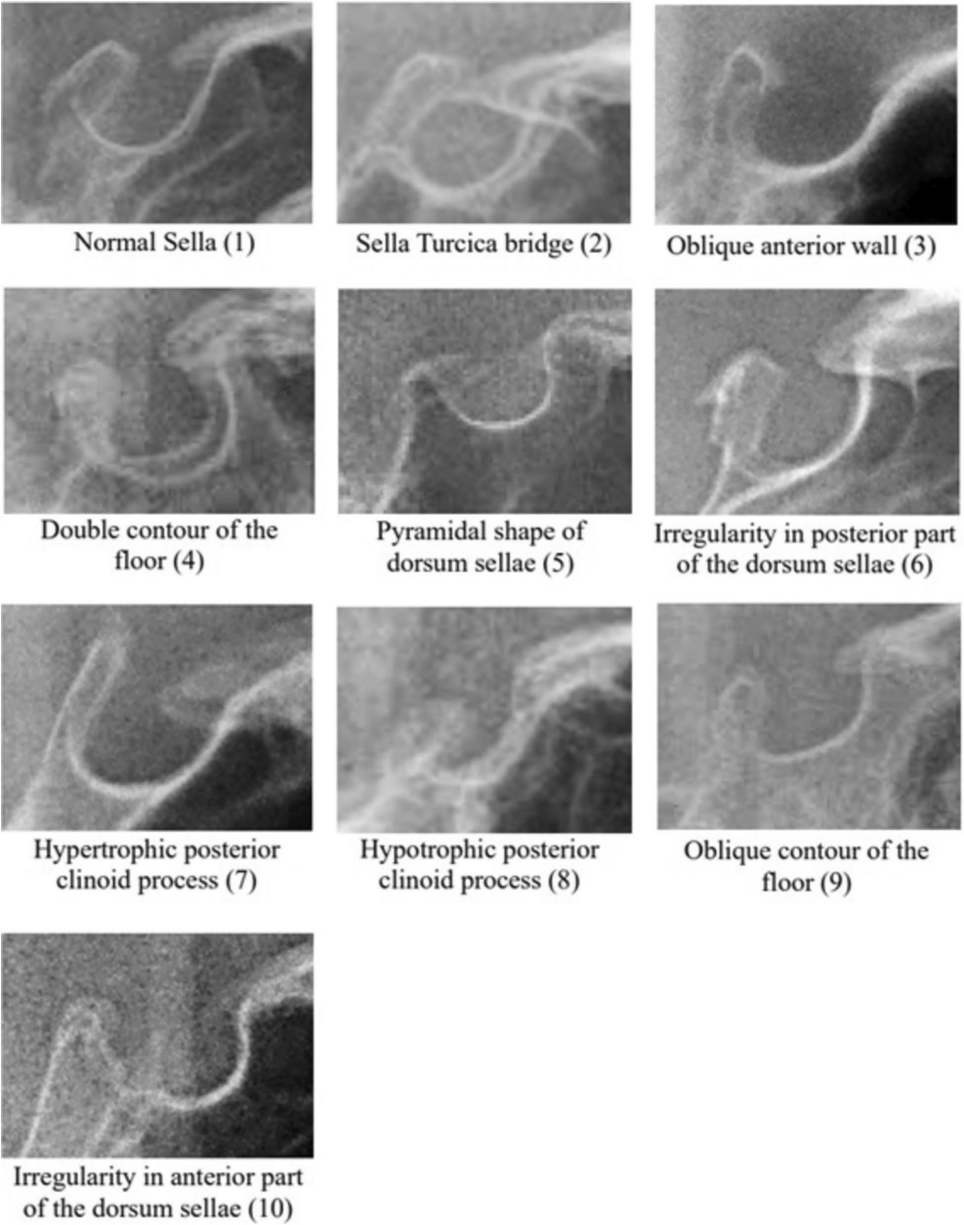
ST shape classified into 10 different forms

Two different classifications were used to analyze ST shapes. Firstly, the tripartite classification—oval (A), round (B), and flat-shaped (C)—was applied according to Gordon et al. from 1923, which was also used in the meta-analysis regarding the morphology of the ST [2, 16–18] (see Fig. 2). For further analysis, the nine-member classification—normal sella (1), sella bridge (2), oblique anterior wall (3), double contour of the floor (4), pyramidal shape of the dorsum sellae (5), irregularity (notching) in the posterior part of dorsum sellae (6), hypertrophic posterior clinoid process (7), hypotrophic posterior clinoid process (8), oblique contour of the floor (9)—was used according to Axelsson and additions by Kucia, as presented in Fig. 3 [8, 15, 18]. Special shapes known as „irregularity in the anterior part of dorsum sellae” (10) were additionally recorded as a tenth shape.

### Cephalometric analysis

Cephalometric measurements were conducted to determine skeletal relations. The skeletal points S (sella), N (nasion), A (maxilla), and B (mandibular) were traced to calculate the ANB angle using the Quick Ceph Studio software tracing feature (San Diego, California, USA). ANB angles between 2° and 4° correspond to class I, angles above 4° to class II, angles below 2° comply with class III.

### Statistical analysis

Statistical analyses were performed using Python software (Python Language Reference, version 3.11.3), using the Pandas library (NumFOCUS, Inc.). SciPy (NumFOCUS, Inc.) was used to perform the statistical tests, whereas Matplotlib (The MathWorks, Inc.) and Seaborn (Michael Waskom; Sphinx and the PyData Theme) were used for plotting and data visualization to evaluate the normality and homogeneity of distribution of data.

Descriptive statistics included the mean, standard deviation, and median values calculated for both gender groups. The bivariate analysis considered the dimension in relation to age. The Shapiro–Wilk test was used for evaluating the distribution of age, ANB, shape, and normality check. The comparison of age distributions between two groups classified by gender was done using the Mann–Whitney *U* test. Additionally, equality of variances was proven by Levene’s test to ensure consistency.

Multivariate analyses, including MANOVA (Multivariate Analysis of Variance) and ANOVA (Analysis of Variance), were conducted to explore relationships between independent variables (age and gender; shape and sagittal malocclusion) and dependent variables (STL length). Various MANOVA methods, such as Wilks’ Lambda, Pillai’s Trace, Hotelling’s Lawley Trace, and Roy’s Greatest Root, were used. MANOVA was used to examine the influence of morphological (size and shape) and demographic (gender) variables, including skeletal class, on ST dimensions (length, depth, anteroposterior diameter). This method was chosen to allow for the simultaneous assessment of multiple dependent variables, providing a comprehensive understanding of how these factors interact to affect ST dimensions. This approach was in line with a similar study by Al-Mohana et al., facilitating direct comparisons [19]. ANOVA was used to compare the means of age and gender in relation to the dimensions of the ST. *P*-values < 0.05 were considered statistically significant. The chi-square test was conducted for correlation tests.

To ensure consistency in the measurements between readers, inter-reader and intra-reader variability for ST measurements and shapes were examined using Intra Class Correlation (ICC). A 95% confidence interval was used.

## Results

### Size of Sella *Turcica*

**Fig. 4:**
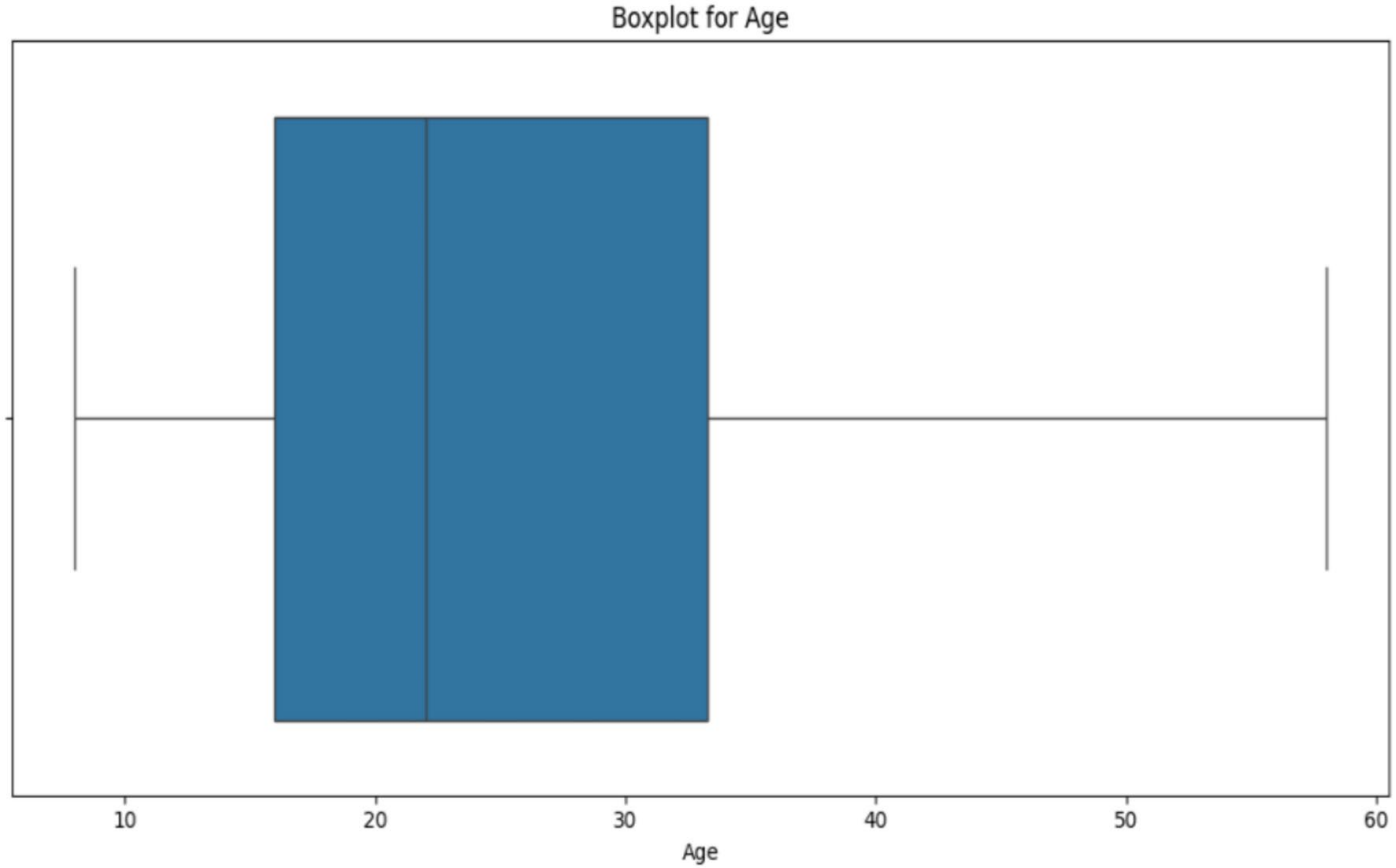
Boxplot depicting age distribution

The linear dimensions of the sella turcica (ST) ranged from 11.1 to 12.9 mm, with a standard deviation of 2.0–2.2 mm. Median lengths spanned from 10.9 to 12.7 mm. The gender distribution in the study was 74.04% females (*n* = 154) and 26.96% males (*n* = 54). Table 1 summarizes the means and standard deviations of ST dimensions. There was no statistically significant difference in the age distribution (Fig. 4) between male and female groups (*p*-value = 0.086). Furthermore, the age variation showed a consistent spread of ages across genders, and no evidence of significant difference in age variability between them.

**Table 1:** Means and standard deviations of ST dimensions

Reader	Length			Depth			Perimeter		
	1	2	3	1	2	3	1	2	3
Mean	12.86	11.08	11.46	8.06	7.93	7.60	34.88	35.89	34.42
SD	2.19	1.98	2.00	1.31	1.31	1.30	4.73	4.40	3.84
Min	7.54	6.12	6.31	4.75	4.79	4.60	25.89	22.36	23.84
25%	11.46	9.78	10.18	7.27	7.06	6.70	31.74	33.24	31.94
50%	12.73	10.95	11.28	8.00	7.87	7.573	34.60	35.40	34.36
75%	14.05	12.19	12.71	8.95	8.59	8.46	37.39	38.54	36.68
Max	23.53	17.51	18.81	12.242	11.24	11.26	65.33	52.70	47.66

The bivariate analysis conducted by all three readers showed a positive correlation between age and the length of the ST, though these correlations were weak (*r* = 0.14–0.26). This suggests a slight tendency for dimensions to increase with age. It should be noted that the linear relationship was not significantly high and might be influenced by other variables. The impact varied across measurements and readers.

When assessing the reliability of the predefined ST measurement techniques, the inter-reader variability among the three readers demonstrated moderate to very good agreement, with an ICC value of 0.8. These high ICC values confirm the consistency and reliability of the measurements taken by the different readers. Intra-reader variability, particularly focused on the average measurements for STDi and STP by one reader, had ICC values of 0.5, indicating moderate consistency and suggesting that the measurement technique is reliable. High ICC values of 0.9, with a confidence interval of 95%, further support a high level of consistency in intra-reader variability.

Furthermore, a post hoc power analysis was conducted to evaluate the statistical power of the study. The analysis revealed a power of approximately 58.5%, indicating that the study is adequately powered to detect medium effect sizes. This finding underscores the reliability of the conclusions drawn from the data, despite the study's preliminary nature.

**Fig. 5:**
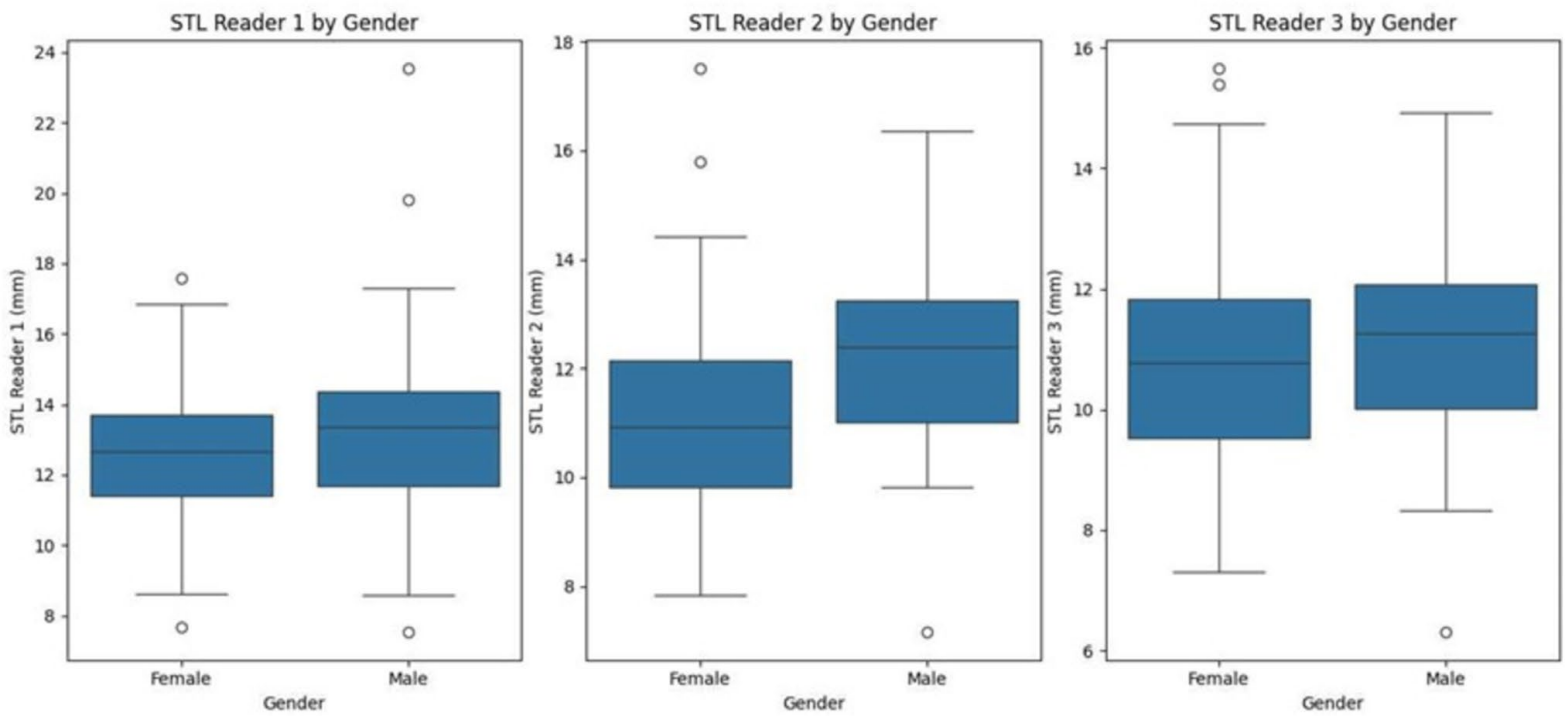
Boxplot diagrams depicting the relationship between gender and ST length. Abbreviation: *ST* Sella turcica, *STL* Sella turcica length

MANOVA analysis showed that both age and gender significantly influenced the length of the sella turcica (STL) collectively (*p* < 0.0001). Moreover, the Wilks' Lambda, Pillai’s Trace, Hotelling-Lawley Trace, and Roy’s Greatest Root tests all indicated a significant overall effect on the combined dependent variables. The interaction between age, gender, and the dimensions of the ST was statistically significant, suggesting that male patients had, on average, a longer ST length compared to female patients, as shown in Fig. 5.

Further, one-way ANOVA analysis indicated that age consistently predicted dimensions across readers (*p* < 0.0015). Age was shown to be a more consistent and significant predictor across all three measurements, although the influence of gender varied.

**Fig. 6:**
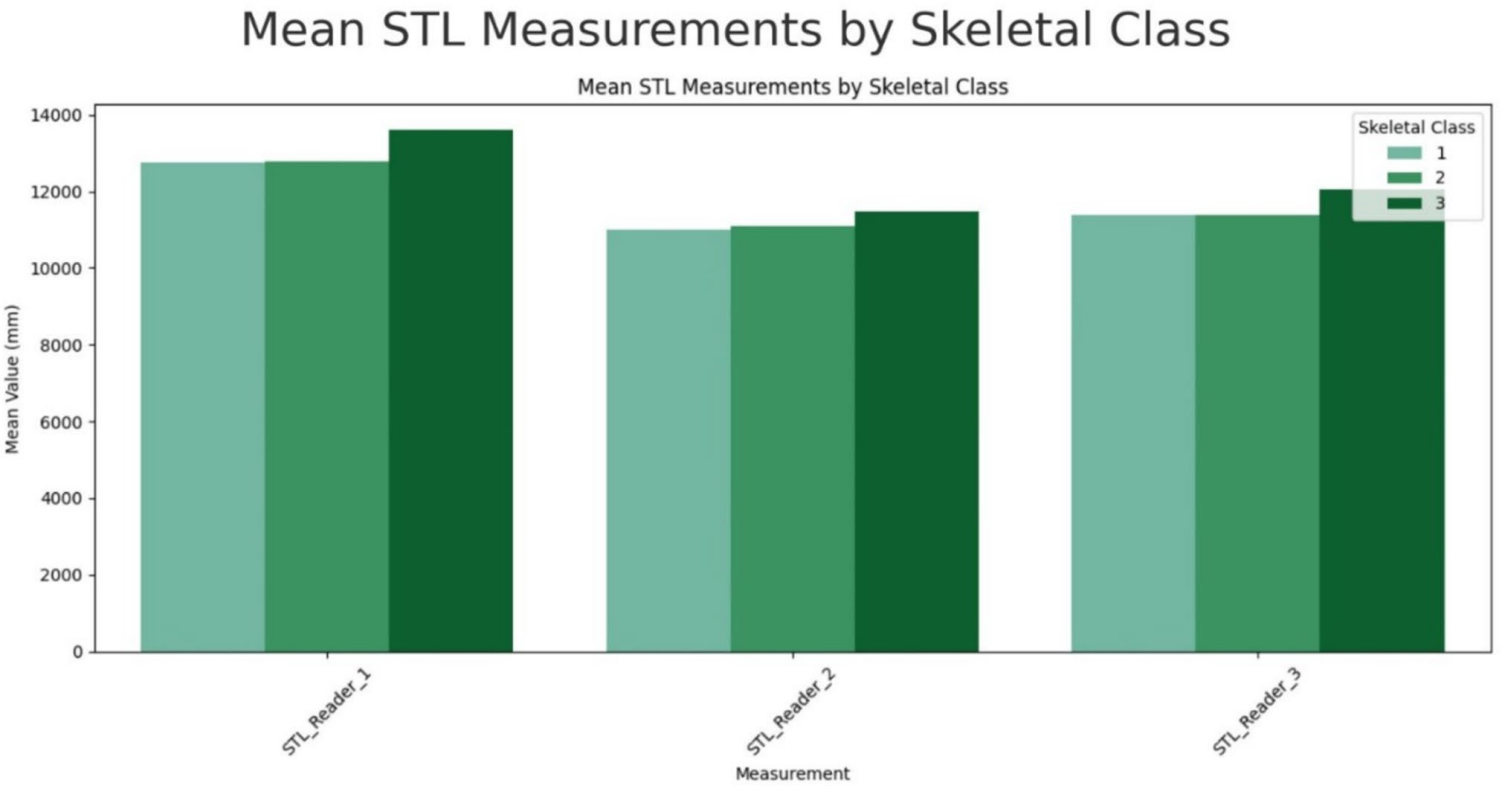
Mean STL measurements by skelettal class

MANOVA analysis of skeletal class influence on size revealed significant differences in ST length, with a *p*-value < 0.0001. Skeletal class III in this study’s population is associated with the largest ST, while class II is only minimally larger than class I sellas, as presented in Fig. 6. Similarly to gender, MANOVA for STDi, STDe, ID, and STP showed no significant results, suggesting that length is the most variable parameter. Shapes by Axelsson and Kucia exhibited significance on size in MANOVA (*p* = 0.0012).

### Shape and bridging of Sella *Turcica*

**Fig. 7:**
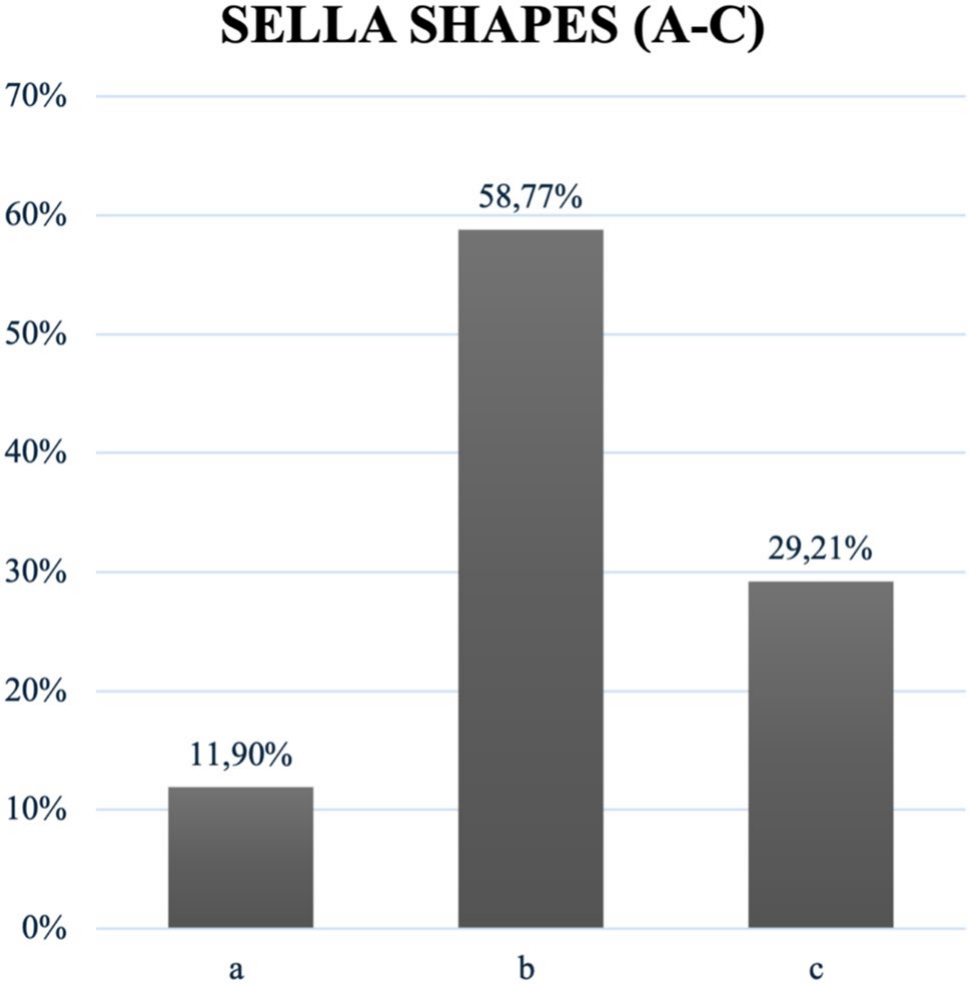
Frequencies of ST shapes round (**a**), oval (**b**) and flat (**c**)

**Fig. 8:**
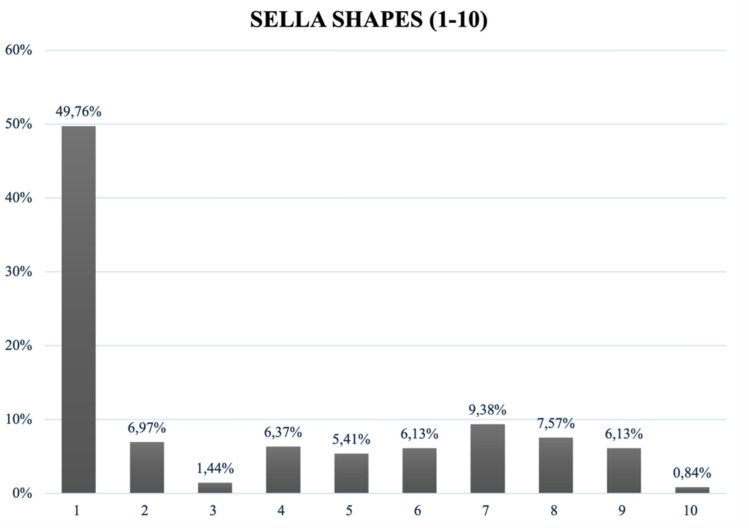
‘Normal Sella’ being the most common sella shape with 49.76% among all 10 shapes

All shapes classified by Axelsson, Kucia, and Gordon were detected in this study’s population [8, 15–18]. A normal sella (shape ‘1’) by Axelsson and Kucia occurred most often—in 49.76% (*n* = 414) of patients. Shape ‘7’ hypertrophic posterior clinoid process appeared with a frequency of 9.38% (*n* = 78), while shape ‘8’ hypotrophic posterior clinoid process occurred in 7.57% (*n* = 53). Shape ‘4’ with double contour of the floor was detected in 6.37% of the cases, whereas shape ‘9’ with oblique contour of the floor together with irregularity in the posterior part of the dorsum sellae (shape ‘6’) was found in 6.13% of the cases. ST bridging was reported as shape ‘2’ in 6.97%. Figure 7 depicts ST shape frequencies following Axelsson and Kucia’s classification. Regarding Gordon’s classification, a round ST (shape ‘b’) was observed most often with 58.77%, followed by a flat sella (shape ‘c’) with 29.21%, and an oval one (shape ‘a’) discovered in 11.90%, as demonstrated in Fig. 8 [16].

The ICC evaluating inter-reader variability among the three different readers showed moderate reliability and agreement among the three raters, with a *p*-value of less than 0.001 in both techniques (shape ‘a–c’ and shape ‘1–10’). ICC assessing intra-reader variability for both shape ‘a–c’ had an ICC value of approximately 0.7 on two different occasions and shape ‘1–10’ also with 0.7 by the same reader three months after the first observations, signifying a good level of consistency in the repeated measurements. These ICC values suggested that the intra-reader variability for both sets of shapes was relatively low, indicating a high degree of reliability in the repeated measurements taken by the same assessor.

ANOVA was used to test whether age influences ST shapes. For shape ‘a–c’ a significant *p*-value of < 0.006 was reached, with even stronger significance shown for shape ‘1–10’ with *p* < 0.0006, indicating clear differences in ST shapes across and plasticity of ST over time.

MANOVA analysis showed no difference in gender on shapes, manifesting no stereotype gender shape of ST. Further MANOVA analysis for shape and size did not show statistical significance either; however, shape alone significantly affects ST dimensions, highlighting the importance of ST morphology in these variations. This study’s population was also tested on skeletal class influence on ST shapes, proclaiming no consistent significance across all readers and both classifications used, tested with chi-square.

### Skeletal class and Sella *Turcica*

In our statistical analysis of ST dimensions across different classes of sagittal malocclusion, the number of observations per class was not uniform. Specifically, our dataset comprised 114 observations for class I (54.8%), 71 for class II (34.2%), and 23 (11.1%) for class III. To ensure a balanced comparison across all classes and maintain the integrity of our analysis, we used an equal number of observations from each class. Given that class III had the fewest observations, with only 23, we matched this number by randomly selecting 23 observations each from classes I and II. This approach allows for a fair and comparative analysis, reflective of the data distribution within our dataset.

This study’s dataset does not indicate significant differences in gender (Chi-square) and age (MANOVA) regarding skeletal classes, indicating their independence. Furthermore, there is no difference in ST dimensions among the classes, with its depth as the only exception reaching the threshold of significance (*p* = 0.05) in a one-way ANOVA. Further investigation is needed to determine any potential meaningful clinical difference. Chi-square test showed no significant association of ST morphology across the skeletal classes. The three-way interaction between age, gender, and skeletal patterns suggests a complex interplay between these variables, although not reaching statistical significance, showing potential subtle influences on ST dimensions or vice versa that merit further investigation.

## Discussion

This study retrospectively analyzed and compared the size, shape, and bridging of ST as well as the skeletal class of Austrian patients using cephalometric lateral radiographs.

To determine the length of the ST, the line from DS to TS was measured, following the approach of Sato and Endo [20]. In comparison to previous studies, as demonstrated by the meta-analysis conducted by Iskra et al., a higher average value was determined [2]. This higher average ST length could be attributed to slight methodological differences, such as calibration variations. On the other hand, it may also indicate an inherently larger ST length in the Austrian population, possibly due to genetic, environmental, or lifestyle factors. To confirm this hypothesis, confirmatory studies with larger sample sizes should be performed throughout Austria. Nevertheless, the high ICC values confirm a strong inter-reader reliability, supporting the validity of our results. The weak positive correlations found between age and ST length (correlation coefficients 0.14–0.26 across readers) were consistent with previous studies, such as the longitudinal study by Axelsson et al., which noted gradual enlargement of the ST length over time from the age of 6 to 21 years [15]. The trend displayed that ST length among males versus females was slightly higher, indicating subtle gender dimorphism in craniofacial parameters [19]. The predominant ST shape “round” (56.25% of subjects) and skeletal class I (54.81%) shown in the meta-analysis also reflected the expected orthodontic population variation [2, 21]. Therefore, the data of the underlying study supported the previously published findings as normative standards. The moderate to near-perfect agreement in ST measurements between readers (ICCs 0.5–0.8) indicated reliable assessment per methods also described by Silvermann and Kisling [16, 22]. This measurement consistency goes in line with previously published ICCs ranging from 0.6 to 0.9 using the same technique [23].

Normal sella shape, being the most common one with 49.76% in this study, reflects existing literature from Turkey (44.4%) as well as most studies in the meta-analysis (55.56%) [2, 24]. Although this suggests that less than half of this population had other shapes than a normal ST, in another Turkish study, only 39.0% showed a normal ST [25]. Bridging (6.97%), being only classified binary here, reflects only half the frequency of worldwide populations (11.34%) and only a mere fraction of Al-Mohana et al.’s bridging frequency of 35.9%. [2, 19]. This discrepancy might be due to a different evaluation by Al-Mohana et al. In their particular study, bridging was defined in greater detail analyzing types of calcifications present. This was because the patients included had relevant syndromes that occur with ST bridging, which were not found in this sample [19]. An oblique anterior wall (shape ‘3’) was the least observed in this study (1.44%), followed by the scientific analysis newly added shape—an irregularity in the anterior part of the dorsum sellae (shape ‘10’)—detected in 0.84%. This new shape will need further studies to confirm its occurrence in other populations as well. Regarding the tripartite classification by Gordon, 58.77% of STs were round-shaped, aligning with worldwide populations as well [2]. Interestingly, the least detected oval shape in this study (11.90%) was the most common shape (48.1%) in Bangladeshi individuals, potentially highlighting ethnological differences [26]. Although the correlation of size and shape showed no significance here, shape was significantly influenced by age. This suggests that while the dimensions of the sella turcica remain relatively consistent, its morphology changes throughout a lifetime. Shape and gender seem to independently influence ST dimensions, strengthening the multifactorial shaping of ST, which needs further investigation. Further research is needed in Austrian and worldwide populations.

Unlike Meyer-Marcotty et al. and Afzal and Fida, who found a significant association between ST morphology and malocclusion classes, the analysis within the sample of this research does not reach significant levels [27, 28]. The predominant morphology in our dataset was the 'oblique anterior wall' (category 'b'), observed in a majority across classes, contrasting with the varied distribution noted by Afzal and Fida, highlighting morphological diversity like 'bridging' and 'doubling of the floor' with specific prevalence rates. The differences in findings could be attributed to sample size, selection criteria, or methodological differences between the analysis of this study and that of Afzal and Fida [28].

### Limitations

Retrospective patient selection was another limitation of this study, as all x-rays obtained were included from a single center for orthodontic purposes. Further, only a third of the selected individuals were male patients, which does not reflect the gender ratio of the Austrian population. A broader expansion of the analysis in the form of a multi-center study would achieve more accurate results for the entirety of Austria. While the study is sufficiently powered to detect medium effect sizes, as indicated by the post hoc power analysis revealing a power of approximately 58.5%, it is important to interpret the findings with some caution. This level of power, while adequate for a preliminary investigation, suggests that larger sample sizes could further validate and expand upon these conclusions. Furthermore, no orthodontic treatment history could be evaluated, possibly weakening the effects of the skeletal class.

Another limiting factor was the type of imaging used. CBCT or CT (computed tomography) imaging technique would have provided more precise results and included an additional reference value. Nevertheless, lateral cephalometric radiographs are the gold standard in orthodontic diagnosis and are most commonly used. Rarely, CBCTs are used in orthodontic treatment planning. Moreover, no study has observed all the above-described parameters before—taking inter- and intra-reader variability into account, thus securing the reliability of measurements.

## Conclusion

In this study, ST size, shape and bridging were analyzed providing first references values of the Austrian population. Further, it shows clear associations of these morphological parameters with demographic data: Age and gender had a significant impact on ST length, contributing to the observed variation in the ST measurements. Age influences ST shapes in two different classifications significantly. Shape exhibits significance on ST size, while there was no significant association found among ST morphology and malocclusion classes.

Although a post hoc power analysis indicated that the study is sufficiently powered to detect medium effect sizes, future research with larger sample sizes is necessary to fully validate these preliminary findings.

The preliminary findings serve as the size reference to aid the clinical interpretation of patient radiographs and diagnosis of pathological anatomical changes in ST. In the future they could potentially contribute to diagnostic and therapeutic strategies in fields such as orthodontics and craniofacial pathology by providing data for AI-based analysis of radiographs. Future studies should aim to enhance the generalizability and clinical utility of these findings by incorporating larger, more diverse samples and employing more advanced imaging techniques such as CBCT.

## Data Availability

The data used in this study, derived from patient records at the Danube Private University Dental Clinic in Krems, Austria, includes sensitive patient information. Due to privacy and confidentiality regulations, the raw data is not publicly available. However, aggregated and anonymized data supporting the findings of this study are available upon reasonable request from the corresponding author.
